# Relationship of Social and Behavioral Characteristics to Suicidality in Community Adolescents With Self-Harm: Considering Contagion and Connection on Social Media

**DOI:** 10.3389/fpsyg.2021.691438

**Published:** 2021-07-13

**Authors:** Eunice Seong, Gahye Noh, Kyung Hwa Lee, Jong-Sun Lee, Sojung Kim, Dong Gi Seo, Jae Hyun Yoo, Hyunchan Hwang, Chi-Hyun Choi, Doug Hyun Han, Soon-Beom Hong, Jae-Won Kim

**Affiliations:** ^1^Division of Child and Adolescent Psychiatry, Department of Psychiatry, Seoul National University Hospital, Seoul, South Korea; ^2^Department of Psychology, Kangwon National University, Chuncheon-si, South Korea; ^3^Department of Psychiatry, Hanyang University Seoul Hospital, Seoul, South Korea; ^4^Department of Psychology, Hallym University, Chuncheon-si, South Korea; ^5^Department of Psychiatry, Seoul St. Mary’s Hospital, The Catholic University of Korea, Seoul, South Korea; ^6^Department of Psychiatry, Chung-Ang University Hospital, Seoul, South Korea; ^7^Department of Psychiatry, SMG-SNU Boramae Medical Center, Seoul, South Korea; ^8^Department of Psychiatry and Institute of Human Behavioral Medicine, Seoul National University College of Medicine, Seoul, South Korea

**Keywords:** adolescent, self-harm, suicidality, social media, contagion, social connection

## Abstract

A close link has been established between self-harm and suicide risk in adolescents, and increasing attention is given to social media as possibly involved in this relationship. It is important to identify indicators of suicidality (i.e., suicide ideation or attempt) including aspects related to contagion in online and offline social networks and explore the role of social media in the relationship between social circumstances and suicidality in young adolescents with self-harm. This study explored characteristics of Korean adolescents with a recent history of self-harm and identified how behavioral and social features explain lifetime suicidality with emphasis on the impact of social media. Data came from a nationwide online survey among sixth- to ninth-graders with self-harm during the past 12 months (*n* = 906). We used χ^2^ tests of independence to explore potential concomitants of lifetime suicidality and employed a multivariate logistic regression model to examine the relationship between the explanatory variables and suicidality. Sensitivity analyses were performed with lifetime suicide attempt in place of lifetime suicidality. 33.9% (*n* = 306) and 71.2% (*n* = 642) reported to have started self-harm by the time they were fourth- and six-graders, respectively; 44.3% (*n* = 400) reported that they have friends who self-harm. Having endorsed moderate/severe forms and multiple forms of self-harm (OR 5.36, *p* < 0.001; OR 3.13, *p* < 0.001), having engaged in self-harm for two years or more (OR 2.42, *p* = 0.001), having friends who self-harm (OR 1.92, *p* = 0.013), and having been bullied at school were associated with an increased odds of lifetime suicidality (OR 2.08, *p* = 0.004). Notably, having posted content about one’s self-harm on social media during the past 12 months was associated with an increased odds of lifetime suicidality (OR 3.15, *p* < 0.001), whereas having seen related content in the same period was not. Sensitivity analyses yielded similar results with lifetime suicide attempt, supporting our findings from the logistic regression. The current study suggests that self-harm may be prevalent from early adolescence in South Korea with assortative gathering. The relationship of vulnerable adolescents’ social circumstances to suicide risk may be compounded by the role of social media. As the role of social media can be linked to both risk (i.e., contagion) and benefit (i.e., social connection and support), pre-existing vulnerabilities alongside SH and what online communication centers on should be a focus of clinical attention.

## Introduction

Adolescent self-harm (SH), the deliberate destruction or alteration of one’s body tissues without suicidal intent, is a growing public health problem, posing adverse emotional, physical, and economic effects on family members (Gratz, 2001; Ferrey et al., 2016). The fifth edition of the Diagnostic and Statistical Manual of Mental Disorders (DSM–5) distinguished between non-suicidal self-injury (NSSI) and suicidal behavior disorder (SBD) and included both as conditions for further study (American Psychiatric Association [APA], 2013). Although SH is conceptually distinguished from suicidal behaviors in the desire and intent involved, still the most disturbing problem with SH lies in its close relationship to suicide, a leading cause of death among adolescents worldwide (World Health Organization [WHO], 2021). A theoretical framework by Thomas Joiner posits that the capability for enacting suicide can be acquired through repeated exposure to painful and provocative experiences such as self-injurious behavior (Joiner, 2007) along with the involvement in other types of violence such as childhood abuse (Serafini et al., 2017). Literature has provided empirical support for this model by not only evidencing that SH history is often a strong correlate of suicide attempt (SA) with suicide ideation (SI), but also demonstrating that an extensive history of SH is associated with an increased risk for suicide; greater versatility (i.e., various methods used) and severity, and longer duration of SH are positively correlated with suicidality (i.e., suicide ideation or attempt) (Nock et al., 2006; Lloyd-Richardson et al., 2007; Klonsky et al., 2013; Turner et al., 2013; Victor and Klonsky, 2014).

Given this link between SH and suicidality, it is increasingly important to identify robust indicators of suicidal risk in community adolescents with SH, especially considering a rising prevalence of SH in this population (Muehlenkamp and Gutierrez, 2007; Tørmoen et al., 2020). While previous studies have attempted to identify behavioral aspects of SH related to a greater risk of suicidality (e.g., severity and number of methods used), this endeavor could be challenging in a group solely consisting of self-injurers because SH history itself often accounts for a large portion of the variance in suicide risk (Klonsky et al., 2013; Turner et al., 2013; Victor and Klonsky, 2014), leaving less variance to other explanatory factors. Another issue may relate to the role of the onset in early adolescence. While SH frequently begins at around age 13 in community adolescents (Stallard et al., 2013; Morey et al., 2017; Gillies et al., 2018), recent trends in adolescent SH including changes in the onset age have not yet been explored much in the general adolescent population (Tørmoen et al., 2020). Previous studies have shown that an earlier onset (i.e., typically at or below age 12) is associated with more frequent, diverse, and severe forms of SH (Ammerman et al., 2018; Muehlenkamp et al., 2018; O’Connor et al., 2018). Despite this potential role of the onset over the course of more pervasive SH, its association with suicidal risk is yet to be sufficiently established especially in a community sample of young adolescents.

Understanding SH in adolescence now necessitates considering the complex social context characterized by rapid interactions on social media (Nesi et al., 2018b) given the drastic increase in social media use affecting adolescent life and the salience of peer influences during this developmental period (Heilbron and Prinstein, 2008; Rideout and Robb, 2018). Social media exerts particular influence over youth’s interpersonal experiences via its unique features (Nesi et al., 2018a) while offering a space to share the narratives and experiences for those who self-harm (Gargiulo and Margherita, 2019). Alarmingly, however, explicit depictions of SH are now easily accessible via popular content-sharing platforms such as Instagram or YouTube (Dyson et al., 2016; Moreno et al., 2016), and young people use Internet searching for information on SH and suicide methods (Mars et al., 2015). Furthermore, with visualness much emphasized in the communication within social media, the involvement of this sensory-specific nature of social media in youth’s behaviors may be intertwined with individual differences that render youth vulnerable to SH, such as sensory processing patterns and avoidant response tendencies particularly in the face of emotional arousal and aversive internal experiences (Chapman et al., 2006; Serafini et al., 2016; Nesi et al., 2018a). Although it may be debatable whether exposure to SH-related content fosters similar behaviors in youth (Shanahan et al., 2019), still being attentive to what teens view and post online is of importance because this may reflect the signs and symptoms of their distress and suicidality (Laffier, 2016; George, 2019). Another aspect of potential risk includes limited guidance on the content and non-compliance with regulations resulting in inadequate protection particularly for vulnerable youth (Dyson et al., 2016; Moreno et al., 2016). George (2019) suggested that the impact of the exposure to SH- and suicide-related posts will be stronger on vulnerable adolescents; adolescents with a history of SH may find these posts more provoking. Nonetheless, only a few studies have investigated a link between exposure to SH posts on social media and engagement in the actual behavior offline; even fewer attempted to link such exposure to suicidal risk among those with a history of SH (Zhu et al., 2016; Arendt et al., 2019).

Relatively underexplored in the area of SH than suicide, the effect of contagion – diffusion of thoughts or behaviors through social network – offers another critical viewpoint (Hawton et al., 2012; Jarvi et al., 2013; Abrutyn et al., 2020). While contagion through direct social ties has been commonly recognized as more influential by means of shared thoughts (Christakis and Fowler, 2009), contagion of suicidal thoughts and behaviors via media has especially emerged as a serious issue to have led the Center for Disease Control to create and distribute guidelines for the reporting of suicide (O’Carroll and Potter, 1994). This is not an overwrought response considering that media coverage of suicide is often associated with the formation of suicide clusters in youth and young adults (Gould et al., 2014). Adolescents can be more susceptible to contagion than adults in general, consistent with the heightened need for social integration in this age group (Reiter et al., 2019), and the impact of contagion in online and offline networks (e.g., via exposure to social media content or direct social ties) may be more pronounced in vulnerable adolescents considering the particular influence of social media and assortative relating involved in their relationship formation (Gould et al., 2003; Joiner, 2003). In this context, it would be imperative to probe the question of how contagion of suicidality manifests in youth sharing a common risk (e.g., SH) and, to begin with, whether exposure to SH content on social media and social ties with other self-injurers can be linked to an increased suicide risk in adolescents who self-harm.

The purpose and hypotheses of this study were as follows. First, we aimed to provide preliminary data on the demographic, social, and behavioral characteristics of community adolescents in South Korea with a recent history of SH. To expand existing knowledge to young adolescents, we specifically focused on a group of sixth- to ninth-graders (typically ages 11–15 years). Next, we aimed to investigate social and behavioral features associated with lifetime suicidality and identify how these features explain lifetime suicidal risk in this group. Based on extant literature, we hypothesized that more severe forms, multiple methods, and longer duration of SH, along with an increased frequency, would be associated with lifetime suicidality and an increased risk. Particularly, assuming the importance of social media content and assortative relating, we hypothesized that exposure to SH posts – viewing and posting of related content – and having friends who self-harm would be associated with lifetime suicidality and an increased risk after controlling for the effect of the aforementioned behavioral aspects of SH.

## Materials and Methods

### Participants and Procedure

The study was based on a nationwide online survey in South Korea. Inclusion criteria consisted of (a) sixth- to ninth-graders; and (b) endorsed at least one act of SH during the past 12 months. Sample was drawn from six stratified districts (i.e., Gyeonggi, Seoul, Gyeongsang, Jeolla, Chungcheong, and Gangwon) according to the estimated proportion in the national population. Participants were recruited via Macromill Embrain^1^, an online research company in South Korea: (1) If parents of eligible sixth- to ninth-graders were enrolled in the firm’s panel, adolescents were contacted by their parents who received an invitation email from the company; (2) If eligible eighth- and ninth-graders were enrolled in the firm’s panel, they were directly contacted via email. The respondents, including the parents in case (1), entered a URL of the survey webpage and initially completed screening questions (Self-Harm Screening, see Supplementary Material 1); those found eligible then completed an online consent form. In case (1), the adolescents additionally completed Self-Harm Screening themselves in a private setting and an online consent form if still found eligible. Adolescents who gave consent answered subsequent questionnaires on demographics, behavioral and social characteristics, and psychological and behavioral constructs. A total of 906 eligible responses were obtained. All participating adolescents and/or parents were informed about the study and signed an online consent form.

The study was approved by the Institutional Review Board (IRB) of Seoul National University Hospital (IRB Number H-1904-093-1027) with a waiver of documentation of consent. A pilot test was conducted among part of the firm’s adolescent panel using Self-Harm Screening to estimate SH appearance. Out of 198 eighth- and ninth-graders that responded, 129 (65.2%) reported at least one act of SH during the past 12 months.

### Assessment

#### Self-Harm Screening

Twenty items were preliminarily devised and used to screen adolescents who have endorsed SH during the past 12 months (see Supplementary Material 1). Based on the review of literature and non-academic sources to identify characteristics of SH behaviors in Korean adolescents, we selected some items from existing measures of SH, such as The Self-Harm Inventory (SHI) (Sansone et al., 1998) as well as rating and adding culturally sensitive items based on clinical observations by an expert group. Depending on whether the behavior was present during the past 12 months, items were answered either “yes” or “no.” For the purpose of this study, SH was explicitly defined as the act of deliberating harming oneself without an intent to commit suicide.

#### Lifetime Suicidality and SA

Lifetime suicidality and SA were assessed using two items from Columbia Suicide Severity Rating Scale (C-SSRS) (Posner et al., 2008): (1) Active SI with some intent to act (i.e., “Have you had suicidal thoughts with some intention of acting on them?”); and (2) Suicidal acts or behavior (i.e., “Have you taken any steps toward making a suicide attempt or preparing to kill yourself (e.g., collecting pills, giving valuables away, writing a suicide note)?”). Lifetime suicidality was regarded as present if item (1) or (2) was answered “yes” and lifetime SA if item (2) was answered “yes.”

#### Socio-Demographics

The sociodemographic variables included sex, grade (year at school), family composition, paternal and maternal education level, and self-reported academic performance.

#### Behavioral Features of SH

Behavioral features of SH were evaluated in five aspects: lifetime frequency, duration, onset, the number of forms in the past 12 months, and severity. Lifetime frequency was categorized as either “five times or more” or “four times or less” based on previous studies (Gargiulo et al., 2019), duration as either “two years or more” or “a year or less,” onset as either “at or below sixth grade (ages 11–12 or below)” or “at or above seventh grade (ages 12–13 or above),” and the number of forms endorsed in the past 12 months as either “multiple” or “single.”

Severity of SH was categorized as either “moderate/severe” or “mild” based on previous studies (Lloyd-Richardson et al., 2007). We first categorized twenty acts listed in Self-Harm Screening according to their severity: “minor,” “moderate/severe,” and “other” (see Supplementary Material 2). We then generated two types based on these categories: “Mild” type consisted of cases with “mild” behaviors only, and “moderate/severe” type included at least one “moderate/severe” behavior. Four cases with “other” behaviors only were excluded from all analyses.

#### Social Circumstances

The social characteristics were investigated as follows: having friends who self-harm, having been bullied at school, having seen content related to one’s SH behaviors on social media during the past 12 months (i.e., “During the past 12 months, have you seen any of the listed behaviors you engaged in on social media such as Facebook, Instagram, Twitter, and/or YouTube?”), and having posted related content on social media during the past 12 months (i.e., “During the past 12 months, have you posted content about any of the listed behaviors you engaged in on social media such as Facebook, Instagram, Twitter, and/or YouTube?”). Items were answered either “yes” or “no.”

### Statistical Analysis

We performed all analyses using R version 3.6.3 for Windows^2^. We first used the chi-squared test of independence to explore potential concomitants of lifetime suicidality. Next, we employed multivariate logistic regression to examine the relationship between explanatory variables and suicidality. Finally, we investigated the model-data fit with the Hosmer–Lemeshow test and computed Nagelkerke’s R^2^ to determine the total variance explained by the explanatory variables included in our model.

## Results

### Sample Characteristics and Associations With Lifetime Suicidality

Table 1 summarizes the demographic, social and behavioral characteristics of the sample (*n* = 902), and the associations between lifetime suicidality and all potential concomitants. Importantly, 33.9% (*n* = 306) reported to have started SH by the time they were fourth-graders: 53.4% (*n* = 126) among the current sixth-graders, 29.2% (*n* = 66), 25.2% (*n* = 58), and 26.7% (*n* = 56) among the seventh-, eighth-, and ninth-graders, respectively. All variables other than paternal and maternal education level and the onset of SH were significantly associated with lifetime suicidality. Contingency coefficients indicated no significant correlation between concomitants, so all variables with a significant association with lifetime suicidality were entered into the regression model.

**TABLE 1:** Sample characteristics and associations with lifetime suicidality (*n* = 902).

**Characteristics**			**SH only (*n* = 764)**	**SH + Suicidality (*n* = 138)**	**χ^2^**	**Unadjusted OR (95% CI)^*a*^**
Socio-demographics	Sex	Male (*n* = 497, 55.1%)	438(88.1%)	59(11.9%)	9.458**	1.00 [reference]
		Female (*n* = 405, 44.9%)	326(80.5%)	79(19.5%)		1.80 (1.25, 2.60)
	Grade (year at school)	6th (*n* = 236, 26.2%)	214(90.7%)	22(9.3%)	12.843**	1.00 [reference]
		7th (*n* = 226, 25.1%)	195(86.3%)	31(17.3%)		1.54 (0.87, 2.79)
		8th (*n* = 230, 25.5%)	188(81.7%)	42(18.3%)		2.16 (1.26, 3.82)
		9th (*n* = 210, 23.3%)	167(79.5%)	43(20.5%)		2.49 (1.45, 4.40)
	Family composition	Both parents (*n* = 844, 93.6%)	724(85.8%)	120(14.2%)	10.581**	1.00 [reference]
		Other (*n* = 58, 6.4%)	40(69.0%)	18(31.0%)		2.72 (1.48, 4.85)
	Father’s education level	≦ High school degree/unknown (*n* = 181, 20.1%)	154(85.1%)	27(14.9%)	4.808	1.05 (0.65, 1.65)
		College degree (*n* = 646, 71.6%)	553(85.6%)	93(14.4%)		1.00 [reference]
		>College degree (*n* = 75, 8.3%)	57(76.0%)	18(24.0%)		1.89 (1.03, 3.30)
	Mother’s education level	≦ High school degree/unknown (*n* = 160, 17.7%)	130(81.3%)	30(18.8%)	2.434	1.41 (0.88, 2.22)
		College degree (*n* = 605, 67.1%)	520(86.0%)	85(14.0%)		1.00 [reference]
		>College degree (*n* = 137, 15.2%)	114(83.2%)	23(16.8%)		1.24 (0.73, 2.02)
	Academic performance	Below average (*n* = 95, 10.5%)	67(70.5%)	28(29.5%)	37.605***	1.83 (1.10, 3.00)
		Average (*n* = 468, 52.0%)	381(81.4%)	87(18.6%)		1.00 [reference]
		Above average (*n* = 339, 37.6%)	316(93.2%)	23(6.8%)		0.32 (0.19, 0.51)
Behavioral features of SH	Frequency (lifetime)	1–4 times (*n* = 474, 52.5%)	417(88.0%)	57(12.0%)	7.739**	1.00 [reference]
		5 times or more (*n* = 428, 47.5%)	347(81.1%)	81(18.9%)		1.71 (1.18, 2.47)
	Severity (past 12 months)	Mild (*n* = 772, 85.6%)	706(91.5%)	66(8.5%)	184.74***	1.00 [reference]
		Moderate/severe (*n* = 130, 14.4%)	58(44.6%)	72(55.4%)		13.20 (8.63, 20.37)
	Number of forms (past 12 months)	Single (*n* = 458, 50.8%)	437(95.4%)	21(4.6%)	80.752***	1.00 [reference]
		Multiple (*n* = 444, 49.2%)	327(73.6%)	117(26.3%)		7.39 (4.63, 12.34)
	Duration	≤1 year (*n* = 393, 43.6%)	350(89.1%)	43(10.9%)	9.619**	1.00 [reference]
		≥2 years (*n* = 509, 56.4%)	414(81.3%)	95(18.7%)		1.86 (1.27, 2.77)
	Onset	≤6th grade (*n* = 642, 71.2%)	542(84.4%)	100(15.6%)	0.068	1.00 [reference]
		≥7th grade (*n* = 260, 28.8%)	222(85.4%)	38(14.6%)		0.93 (0.61, 1.38)
Social circumstances	Having friends who self-harm	No (*n* = 502, 55.7%)	466(92.8%)	36(7.2%)	56.306***	1.00 [reference]
		Yes (*n* = 400, 44.3%)	298(74.5%)	102(25.5%)		4.41 (2.96, 6.71)
	Having been bullied at school	No (*n* = 705, 78.2%)	635(90.1%)	70(9.9%)	69.954***	1.00 [reference]
		Yes (*n* = 197, 21.8%)	129(65.5%)	68(34.5%)		4.77 (3.25, 7.01)
	Having seen SH content on social media (past 12 months)	No (*n* = 623, 69.1%)	572(91.8%)	51(8.2%)	76.877***	1.00 [reference]
		Yes (*n* = 279, 30.9%)	192(68.8%)	87(31.2%)		5.07 (3.47, 7.47)
	Having posted SH content on social media (past 12 months)	No (*n* = 797, 88.4%)	712(89.3%)	85(10.7%)	110.42***	1.00 [reference]
		Yes (*n* = 105, 11.6%)	52(49.5%)	53(50.5%)		8.50 (5.45, 13.30)

*SH, Self-harm.*

^*a*^The reference group is SH only (*n* = 764).

*p < 0.05, **p < 0.01, ***p < 0.001.

### Multivariate Logistic Regression for Lifetime Suicidality

Table 2 presents the relationship between the explanatory variables and lifetime suicidality in a logistic regression model. Having endorsed at least one moderate/severe form of SH during the past 12 months compared to mild forms only (OR 5.36, *p* < 0.001) and multiple forms of SH compared to a single form (OR 3.13, *p* < 0.001), having engaged in SH for two years or more compared to a year or less (OR 2.42, *p* = 0.001), having friends who self-harm (OR 1.92, *p* = 0.013), having been bullied at school (OR 2.08, *p* = 0.004), and having posted content about one’s SH on social media during the past 12 months (OR 3.15, *p* < 0.001) were associated with an increased odds of lifetime suicidality. However, the effect of five times or more SH compared to four times or less and having seen content related to SH one engaged in on social media during the past 12 months was not significant (OR 1.10, *p* = 0.688; OR 1.54, *p* = 0.101).

**TABLE 2:** Multivariate logistic regression for lifetime suicidality (*n* = 902).

**Characteristics**		**SH + Suicidality (*n* = 138)**	
			**Adjusted OR (95% CI)^*a*^**
Socio-demographics	Sex	Male	1 [reference]
		Female	1.61* (1.01, 2.60)
	Grade (year at school)	6th	1 [reference]
		7th	1.33 (0.65, 2.79)
		8th	1.69 (0.83, 3.52)
		9th	2.08* (1.03, 4.31)
	Family composition	Both parents	1 [reference]
		Other	1.52 (0.65, 3.43)
	Academic performance	Below average	1.01 (0.50, 1.97)
		Average	1 [reference]
		Above average	0.39** (0.22, 0.69)
Behavioral features of SH	Frequency (lifetime)	1–4 times	1 [reference]
		5 times or more	1.10 (0.68, 1.79)
	Severity (past 12 months)	Mild	1 [reference]
		Moderate/severe	5.36*** (3.06, 9.49)
	Number of forms (past 12 months)	Single	1 [reference]
		Multiple	3.13*** (1.78, 5.69)
	Duration	≤1 year	1 [reference]
		≥2 years	2.42** (1.44, 4.18)
Social circumstances	Having friends who self-harm	No	1 [reference]
		Yes	1.92* (1.15, 3.22)
	Having been bullied at school	No	1 [reference]
		Yes	2.08** (1.27, 3.40)
	Having seen SH content on social media (past 12 months)	No	1 [reference]
		Yes	1.54 (0.92, 2.56)
	Having posted SH content on social media (past 12 months)	No	1 [reference]
		Yes	3.15*** (1.70, 5.84)

*SH, Self-harm.*

^*a*^The reference group is SH only (*n* = 764).

*p < 0.05, **p < 0.01, ***p < 0.001.

The regression model provided a satisfactory model-data fit (Hosmer–Lemeshow test *p* = 0.934), and the explanatory variables accounted well for the total variance in lifetime suicidality (Nagelkerke R^2^ = 0.462).

### Sensitivity Analyses With Lifetime SA

For results in terms of an outcome most often explored in existing literature (e.g., SA), we conducted sensitivity analyses by repeating the analytic procedures using a subset of data. Data were on cases with SH only (*n* = 764) and those with both SH and SA (*n* = 82). Fifty-six participants reporting lifetime SI without SA [i.e., item (1) but (2) answered “yes” from Lifetime suicidality and SA] were excluded. The reference group was SH only (*n* = 764), and the dependent variable in the logistic regression was SA (*n* = 82).

All demographic, social, and behavioral characteristics but the onset of SH were significantly associated with lifetime SA (see Table 3). The following results were obtained from a multivariate logistic regression for lifetime SA with the same set of explanatory variables used in Table 2 (see Table 4). The relationship of social and behavioral features to lifetime SA was overall similar to the results from Table 2. Having endorsed at least one moderate/severe form of SH during the past 12 months compared to mild forms only (OR 6.36, *p* < 0.001) and multiple forms of SH compared to a single form (OR 6.57, *p* < 0.001), having engaged in SH for two years or more compared to a year or less (OR 2.73, *p* = 0.005), having friends who self-harm (OR 2.43, *p* = 0.015), having been bullied at school (OR 1.96, *p* = 0.041), and having posted content about one’s SH on social media during the past 12 months (OR 3.87, *p* < 0.001) were associated with an increased odds of lifetime SA. Consistent with the results from Table 2, the effect of five times or more SH compared to four times or less and having seen content related to SH one engaged in on social media during the past 12 months was not significant (OR 1.19, *p* = 0.597; OR 1.37, *p* = 0.379).

**TABLE 3:** Characteristics of the subsample and associations with lifetime SA (*n* = 846).

**Characteristics**			**SH only (*n* = 764)**	**SH + SA (*n* = 82)**	**χ^2^**	**Unadjusted OR (95% CI)^*a*^**
Socio-demographics	Sex	Male (*n* = 469, 55.4%)	438(93.4%)	31(6.6%)	10.651**	1.00 [reference]
		Female (*n* = 377, 44.6%)	326(86.5%)	51(13.5%)		2.20 (1.39, 3.56)
	Grade (year at school)	6th (*n* = 226, 26.7%)	214(94.7%)	12(5.3%)	9.960*	1.00 [reference]
		7th (*n* = 213, 25.2%)	195(91.5%)	18(8.5%)		1.64 (0.77, 3.60)
		8th (*n* = 214, 25.3%)	188(87.9%)	26(12.1%)		2.44 (1.22, 5.18)
		9th (*n* = 193, 22.8%)	167(86.5%)	26(13.5%)		2.75 (1.37, 5.84)
	Family composition	Both parents (*n* = 794, 93.9%)	724(91.2%)	70(8.8%)	9.768**	1.00 [reference]
		Other (*n* = 52, 6.1%)	40(76.9%)	12(23.1%)		3.12 (1.50, 6.08)
	Father’s education level	≦ High school degree/unknown (*n* = 175, 20.7%)	154(88.0%)	21(12.0%)	14.047***	1.64 (0.93, 2.81)
		College degree (*n* = 599, 70.8%)	553(92.3%)	46(7.7%)		1.00 [reference]
		>College degree (*n* = 72, 8.5%)	57(79.2%)	15(20.8%)		3.17 (1.62, 5.95)
	Mother’s education level	≦ High school degree/unknown (*n* = 151, 17.8%)	130(86.1%)	21(13.9%)	6.983*	1.91 (1.08, 3.30)
		College degree (*n* = 564, 66.7%)	520(92.2%)	44(7.8%)		1.00 [reference]
		>College degree (*n* = 131, 15.5%)	114(87.0%)	17(13.0%)		1.77 (0.95, 3.16)
	Academic Performance	Below average (*n* = 88, 10.4%)	67(76.1%)	21(23.9%)	33.675***	2.49 (1.38, 4.39)
		Average (*n* = 429, 50.7%)	381(88.8%)	48(11.2%)		1.00 [reference]
		Above average (*n* = 329, 38.9%)	316(96.0%)	13(4.0%)		0.33 (0.17, 0.60)
Behavioral features of SH	Frequency (lifetime)	1–4 times (*n* = 447, 52.8%)	417(93.3%)	30(6.7%)	8.915**	1.00 [reference]
		5 times or more (*n* = 399, 47.2%)	347(87.0%)	52(13.0%)		2.08 (1.30, 3.37)
	Severity (past 12 months)	Mild (*n* = 738, 87.2%)	706(95.7%)	32(4.3%)	184.74***	1.00 [reference]
		Moderate/Severe (*n* = 108, 12.8%)	58(53.7%)	50(46.3%)		18.84 (11.28, 31.98)
	Number of forms (past 12 months)	Single (*n* = 443, 50.8%)	437(98.6%)	6(1.4%)	71.881***	1.00 [reference]
		Multiple (*n* = 403, 49.2%)	327(81.1%)	76(18.9%)		16.49 (7.68, 43.28)
	Duration	≤1 year (*n* = 375, 44.3%)	350(93.3%)	25(6.7%)	6.439*	1.00 [reference]
		≥2 years (*n* = 471, 55.7%)	414(87.9%)	57(12.1%)		1.92 (1.19, 3.19)
	Onset	≤6th grade (*n* = 606, 71.6%)	542(89.4%)	64(10.6%)	1.507	1.00 [reference]
		≥7th grade (*n* = 240, 28.4%)	222(92.5%)	18(7.5%)		0.69 (0.39, 1.17)
Social circumstances	Having friends who self-harm	No (*n* = 483, 57.1%)	466(96.5%)	17(3.5%)	47.375***	1.00 [reference]
		Yes (*n* = 363, 42.9%)	298(82.1%)	65(17.9%)		5.93 (3.48, 10.65)
	Having been bullied at school	No (*n* = 674, 79.7%)	635(94.2%)	39(5.8%)	55.618***	1.00 [reference]
		Yes (*n* = 172, 20.3%)	129(75.0%)	43(25.0%)		5.41 (3.37, 8.72)
	Having seen SH content on social media (past 12 months)	No (*n* = 598, 70.7%)	572(95.7%)	26(4.3%)	64.51***	1.00 [reference]
		Yes (*n* = 248, 29.3%)	192(77.4%)	56(22.6%)		6.38 (3.93, 10.61)
	Having posted SH content on social media (past 12 months)	No (*n* = 757, 89.5%)	712(94.1%)	45(5.9%)	111.46***	1.00 [reference]
		Yes (*n* = 89, 10.5%)	52(58.4%)	37(41.6%)		11.19 (6.65. 18.87)

*SH, Self-harm; SA, Suicide attempt.*

^*a*^The reference group is SH only (*n* = 764).

*p < 0.05, **p < 0.01, ***p < 0.001.

**TABLE 4:** Multivariate logistic regression for lifetime SA (*n* = 846).

**Characteristics**		**SH + SA (*n* = 82)**	
			**Adjusted OR (95% CI)^*a*^**
Socio-demographics	Sex	Male	1 [reference]
		Female	1.75 (0.93, 3.34)
	Grade (year at school)	6th	1 [reference]
		7th	1.51 (0.58, 4.09)
		8th	2.02 (0.78, 5.47)
		9th	2.58 (1.02, 6.93)
	Family composition	Both parents	1 [reference]
		Other	1.56 (0.53, 4.33)
	Academic performance	Below average	1.17 (0.49, 2.69)
		Average	1 [reference]
		Above average	0.37* (0.16, 0.79)
Behavioral features of SH	Frequency (lifetime)	1–4 times	1 [reference]
		5 times or more	1.19 (0.63, 2.27)
	Severity (past 12 months)	Mild	1 [reference]
		Moderate/severe	6.36*** (3.18, 12.96)
	Number of forms (past 12 months)	Single	1 [reference]
		Multiple	6.57*** (2.73, 18.53)
	Duration	≤1 year	1 [reference]
		≥2 years	2.73** (1.37, 5.67)
Social circumstances	Having friends who self-harm	No	1 [reference]
		Yes	2.43* (1,20, 5.07)
	Having been bullied at school	No	1 [reference]
		Yes	1.96* (1.02, 3.73)
	Having seen SH content on social media (past 12 months)	No	1 [reference]
		Yes	1.37 (0.68, 2.74)
	Having posted SH content on social media (past 12 months)	No	1 [reference]
		Yes	3.87*** (1.78, 8.46)

*SH, Self-harm; SA, Suicide attempt.*

^*a*^The reference group is SH only (*n* = 764).

*p < 0.05, **p < 0.01, ***p < 0.001.

## Discussion

In this community-based study, we explored characteristics of Korean young adolescents with SH and identified their relationship to lifetime suicidality with emphasis on the impact of social media. In addition to comprehensively address behavioral components of SH, we addressed exposure to SH-related social media content and social connection with other self-injurers as two processes possibly involved in suicidality among those sharing a common risk, namely SH. Notably, different results were found between “posting” and “seeing” content about one’s SH during the past 12 months, with only having posted content associated with an increased risk of lifetime suicidality and SA when controlling for other behavioral and social features. This may be understood as rendering support to the proposed impact of social media posts while suggesting that specific mechanisms that underlie more “active” and rather “passive” exposure are related to different outcomes. As concerns direct social ties to other self-injurers, slightly less than half of the adolescents in this study reported having friends who self-harm, with this condition also linked to an increased risk of suicidality and SA history.

### Behavioral Features of SH and Suicidality

For the most part, the relationship between behavioral features of SH and suicidality in our young adolescent sample corroborated previous findings (Nock et al., 2006; Lloyd-Richardson et al., 2007; Jacobson et al., 2008; Duarte et al., 2020); more severity, a greater number of forms, and longer duration of SH were associated with an increased risk. Though limited in making predictive inferences, the current study demonstrates that more practice with and exposure to SH in terms of time (i.e., duration), means (i.e., the number of forms) and severity are associated with a greater risk of suicidality in community adolescents, consistent with the notion that the capability for suicide can be acquired through self-inflicted injury, and that past experiences with self-injury can facilitate the acts of serious lethality (e.g., suicidal behaviors) (Joiner, 2007; Van Orden et al., 2010). Being a correlate of suicidality and SA history, however, lifetime frequency of SH explained neither of these outcomes in our regression models. Based on the view that repeated SH and subsequent habituation to the fear and pain involved in self-injurious behaviors provide the groundwork for suicide crises (Joiner, 2007), this somewhat undermined importance of frequency may not be readily interpretable. Although frequency of SH has often been noted as an indicator of suicide risk (Victor and Klonsky, 2014), the association of this feature with suicidal behaviors is not as robust as that of other features of SH, with frequency often regarded more as a proxy for other measures (e.g., severity and duration) (Nock et al., 2006; Muehlenkamp and Gutierrez, 2007; Hamza et al., 2012; Turner et al., 2013; Mars et al., 2019).

An “early onset” of SH (at or below ages 11–12) was neither a significant correlate of suicidality nor of SA history in the present study. When compared to Brager-Larsen et al. (2021) in which the sample consisted of psychiatric outpatients, this result may be suggestive of the heightened importance of SH onset in suicidal risk among those with clinically significant symptoms; interacting with psychological distress (e.g., depression), SH started at an early age may possibly develop into more pervasive forms of SH which, in turn, aggravate the risk for suicidality. It may be that, for those without clinically meaningful symptoms, onset maintains a rather distal relationship to suicidality mainly via other aspects of SH (e.g., lifetime frequency, versatility, and severity) than being directly linked to the risk throughout the trajectory of SH. However, to valorize current findings and further clarify the role of the onset over the course of SH, more studies with longitudinal designs and different age groups would be needed. Given that participation was limited to sixth- to ninth-graders, the reduced importance of the onset in the current study can also be explained by the restricted age range of the sample, posing two issues to be considered: the opt-out of individuals with a typical or later onset and relatively short duration of SH. It is still worth noting, though, that over one-third and two-thirds of the sample reported to have started SH by the time they were fourth- and sixth-graders (ages 9–10 and ages 11–12), respectively. This may be in line with a previous report on the decrease in the age at SH onset among hospital-treated young adults (Griffin et al., 2018) while adding to the existing literature underscoring the importance of more timely prevention efforts made upon age 12 or below before SH becomes established (Stallard et al., 2013).

### Social Media in the Relationship Between Adolescents’ Social Circumstances and Suicidality

A noticeable finding regarding exposure to social media posts was that having posted content about one’s SH during the past 12 months was associated with an increased risk of lifetime suicidality and SA when controlling for other behavioral and social features, while merely having seen related content in the same period did not maintain its association with suicidality risk after adjusting for other variables. A similar attempt to differentiate between “posting” and “seeing” content has recently been made by Swedo et al. (2020) in a study on youth suicide clusters. Posting suicide-related content was overall associated with an increased odds of both SI and SA, while seeing related posts was only associated with an increased odds of SI when confounding variables were adjusted (Swedo et al., 2020). Results from the present study support the idea that more active involvement, such as creating and posting content, likely occurs among those at higher risk on the spectrum of suicidality (Swedo et al., 2020). This involvement may be differentiated in some respects from a typical way of “being active” on social media (e.g., sharing photos or updating one’s status) which is often associated with fewer psychological symptoms, possibly via the role of social support (Frison and Eggermont, 2016; Thorisdottir et al., 2019). An explanation to this might be that producing and posting SH or SA content are motivated by different thought processes and could be read more as a “cry for help” or “a rehearsal of suicide plan” (Swedo et al., 2020). Future studies will need to differentiate between specific behaviors on social media (e.g., browsing, reposting, and watching others’ posts), the extent to which individuals are psychologically and socially engaged in these behaviors, and the degree of intentionality involved in the exposure to potentially harmful content to better understand how social media can add to the risk of SH and suicide in vulnerable adolescents (Arendt et al., 2019).

That a little less than half of the participants responded that they have friends who self-harm may not only reflect that relationship is an important area that unites adolescents with SH (Gargiulo, 2020) but also that assortative relating is common among self-injurers from an early age. It has been suggested that assortative relating alone may not be sufficient to explain the contagion of suicidal symptoms in adolescents and young adults and that the shared risk factors that pre-exist need to be considered to properly weigh its impact (Hawton et al., 2020). To some degree, an increased risk in adolescent self-injurers could be reflected in their social ties as documented in the connection between peer’s SH and an increased risk of lifetime suicidality in our study. Taken together with the literature, this poses further questions to be explored including whether suicidality contagion can be exacerbated with SH as a shared risk while considering the role of social media. Specifically, two routes could be considered: with social media offering a channel for assortative gathering based on the commonalities (e.g., SH), enabling online interactions among those who are pulled together with similar others more active; and by providing information on suicidal behaviors and making discussion on the topic more feasible, thereby habituating youth to the idea of suicide (Joiner, 2007). Either way, the difference observed between viewing and posting of SH content in explaining suicidality may also relate to different degrees of involvement in these dynamics in social media networks, with posting content about one’s own SH reflecting more involvement than observing related content.

The relationship of adolescent bullying victimization experience to suicidality history can further be understood with thwarted belongingness as a psychological and interpersonal construct of suicidal desire (Joiner, 2007; Van Orden et al., 2010). Documenting that having been a victim of bullying is associated with an elevated risk for lifetime suicidality in adolescents with SH, the present study suggests that experience of frustrated belongingness is somehow linked to a heightened risk among adolescents displaying a certain level of capability for self-injury, in accordance with the mounting evidence that bullying victimization in childhood and adolescence, a typical form of social exclusion, is associated with various forms of psychological distress, SH, and suicidality over the life course (Fisher et al., 2012; Winsper et al., 2012; Lereya et al., 2013, 2015; Takizawa et al., 2014; Geoffroy et al., 2016). The sequence of the events is unknown in our study, yielding the result open to a number of viable interpretations. While a frustrated need to belong could have instigated suicide desire among those with an accrued behavioral capability of self-injury, it is also possible that victims of bullying turned more to SH as a means to relieving interpersonal stress and psychological pain and, with repetition of SH, have developed suicidal thoughts and behaviors. Alternatively, pre-existing risks (e.g., maladaptive family experiences or impulsity) may have rendered youth more prone to both social exclusion and engagement in SH which, taken together, contributed to higher suicidal risk (Lereya et al., 2013).

Regardless of the form it takes, such relationship between bullying victimization, SH, and suicidality in youth can further be compounded by social media both in a harmful and protective way (Hawton et al., 2020). Adolescents perceiving a lack in meaningful real-world social contact could rely more on online interactions (Gross, 2009) which may, on the one hand, add to the risk by facilitating active discussion of suicidal thoughts and behaviors, but on the other hand, put the brakes on severe thoughts and behaviors by offering a sense of belongingness (Joiner, 2007; Van Orden et al., 2010). The benefit social media can provide in terms of online social connection appears more valid considering that interpersonal connection and support can often be powerful enough to buffer against suicidality posed by other risks (e.g., family adversities) (Cho and Haslam, 2010; Forster et al., 2020). It should also be noted that often young people who access SH content online have been engaging in SH already and that social media, along with other online environments, can be a protected space for these people where the experience of SH can emerge and be shared (Gargiulo and Margherita, 2019; Lavis and Winter, 2020). Insofar as its roles can be linked to both risk (i.e., contagion) and benefit (i.e., social connection and support), social media itself is not necessarily a risk for adolescents with SH, but the pre-existing vulnerabilities alongside SH and what online communication centers on would be a focus of clinical attention. Further research is warranted to elucidate when and how relationship and communication on social media that cluster around SH either aggravate or buffer suicide risk, along with its specific mechanism (e.g., alterations in norms and strengthened cohesion), in order to accurately assess the impact of contagion and connection in social media for at-risk adolescents and identify points for strategic interventions.

### Strengths and Limitations

The present study furthered previous understanding of social contagion in adolescent SH by linking two possible means of contagion in previous literature (i.e., social media exposure and peer’s SH) to suicidality in young adolescents with a recent SH history. Differing outcomes linked to more “active” engagement compared to rather a “passive” exposure to SH posts were discussed with regard to the dynamics in social media networks as well as the individual intention involved and signs of distress. A considerable proportion of adolescent self-injurers reporting peer’s SH and its connection with an increased risk of lifetime suicidality indicate the prevalence and potential importance of assortative gathering in youth with SH. While social media is possibly involved in assortative relating in this vulnerable population given its transformative role in peer relationship, and the spread of SH and suicidality via potentially provoking posts, it can also be a means of providing social connection and support especially for those in lack of a meaningful social contact. Additionally, by including sixth- and seventh-graders (ages 11–13) reporting a history of SH proportional to eighth- and ninth-graders (ages 13–15) in the sample, this study expanded previous knowledge to a group of adolescents both above and under the average age at SH onset. More pervasive SH in terms of time (i.e., duration), means (i.e., the number of forms) and severity was associated with a greater risk of lifetime suicidality in young community adolescents, in accordance with previous findings from other samples. That more than two-thirds of the sample have started SH by ages 11–12 indicates that SH could be prevalent in Korean adolescents from an earlier age than previously known, suggesting that clinical attention is required.

Several limitations and shortcomings of this study are discussed as follows. First, due to the cross-sectional nature of the study design, it is difficult to conclude that some explanatory variables (e.g., bullying victimization and exposure to SH via social media posts) predict subsequent risk of suicidality and SA. Studies with longitudinal design are needed to further strengthen the findings from this study. Another methodological limitation may involve the measurement of the explanatory variables: based on the reference point of some binary variables (e.g., lifetime frequency of SH), results may be open to changes. Also, social circumstances of adolescents (e.g., friends who self-harm) could be quantitatively measured for more informative results in future studies. Next, we did not account for the role of SI and separated it neither as an associate nor as an outcome. SI is often the strongest correlate of SA history among self-injurers while explaining an increased risk of SA in adolescent self-injurers (Victor and Klonsky, 2014; Duarte et al., 2020). In community adolescents, augmented SI is also apparent in those with both NSSI and SA than those only with NSSI (Muehlenkamp and Gutierrez, 2007). Given this pronounced role of SI, future research will need to explore how SI interacts with features investigated in the present study especially throughout contagion and connection on social media to explain SA. Finally, although consisting of community adolescents, neither clinical features nor psychological correlates of the present sample were identified. While the co-occurrence of SH and SA is common in both clinical and non-clinical samples of adolescents (Nock et al., 2006; Taliaferro et al., 2012), the two often differ in suicidal risk as well as in behavioral features of SH (e.g., frequency and number of methods) (Jacobson and Gould, 2007; Hamza et al., 2012; Duarte et al., 2020). In this regard, future studies will benefit from taking into account psychological correlates (e.g., depressive symptoms) to more accurately assess the relationship between the social and behavioral aspects of SH and suicidality in community adolescents.

## Data Availability Statement

The raw data supporting the conclusions of this article will be made available by the authors, without undue reservation.

## Ethics Statement

The studies involving human participants were reviewed and approved by the Institutional Review Board (IRB) of Seoul National University Hospital (IRB Number H-1904-093-1027) with a waiver of documentation of consent. Written informed consent to participate in this study was provided by the participants’ legal guardian/next of kin.

## Author Contributions

J-WK, J-SL, SK, DS, C-HC, DH, and JY were involved in obtaining research funding and designing the survey. J-SL, SK, and DS developed Self-Harm Screening items. HH contributed to establishing the survey protocol and collecting the data. J-WK, ES, KL, and S-BH formulated research questions. ES undertook data analyses and wrote the manuscript with GN. KL contributed to editing the manuscript. J-WK supervised the study. All authors contributed to the article and approved the submitted version.

## Conflict of Interest

The authors declare that the research was conducted in the absence of any commercial or financial relationships that could be construed as a potential conflict of interest.

## Footnotes

1 https://embrain.com

2 https://cran.r-project.org/

## Abbreviations

SH: Self-harm
SA: Suicide attempt
SI: Suicide ideation.
