# Supplementary material for: Relationship of Social and Behavioral Characteristics to Suicidality in Community Adolescents With Self-Harm: Considering Contagion and Connection on Social Media
Source: Front Psychol. 2021 Jul 13;12:691438. doi: 10.3389/fpsyg.2021.691438 (PMC8315269; doi:10.3389/fpsyg.2021.691438)
Supplement: Supplementary file 1 [file Data_Sheet_1.PDF]

Supplementary Material 1

Supplementary Material 1- 1 Self-Harm Screening

This questionnaire asks about various self-harm behaviors. Please read each statement and mark “yes” if you (or your children) have engaged in such behavior at least once during the past year or “no” if not. (Self-harm behavior refers to the act of deliberating harming oneself without the intent to commit suicide.)

1	Overdosed on drugs	Yes	No
2	Cut my body with sharp objects		
3	Hit my body (e.g. hitting my body, such as the head, hard with my hands)		
4	Banged head against a wall, desk, etc.		
5	Hit things hard with a fist		
6	Scratched my body		
7	Cut or carved something onto skin using a knife		
8	Pulled my hair out		
9	Picked or pinched my wound		
10	Burnt skin with fire (using cigarette, match, or other hot objects)		
11	Stuck objects underneath fingernails or into skin		
12	Bit parts of my body (e.g. mouth, lips, etc.)		
13	Scratched skin until it left scars		
14	Picked or peeled off skin		
15	Stabbed my body with sharp or pointed objects		
16	Cut holes in my body (e.g., not ear piercings or body piercings that are meant to enhance beauty, but to make holes to harm myself)		
17	Slit or cut my body with sharp objects		
18	Carved words or symbols onto body		
19	Strangled my neck		
20	Engaged in bloodletting (drained blood from my body)		
