# Supplementary material for: Relationship of Social and Behavioral Characteristics to Suicidality in Community Adolescents With Self-Harm: Considering Contagion and Connection on Social Media
Source: Front Psychol. 2021 Jul 13;12:691438. doi: 10.3389/fpsyg.2021.691438 (PMC8315269; doi:10.3389/fpsyg.2021.691438)
Supplement: Supplementary file 2 [file Data_Sheet_2.PDF]

Supplementary Material 2

Supplementary Material 2- 1 Severity categorization of Self-Harm Screening

Mild	
3	Hit my body (e.g. hitting my body, such as the head, hard with my hands)
4	Banged head against a wall, desk, etc.
5	Hit things hard with a fist
6	Scratched my body
8	Pulled my hair out
9	Picked or pinched my wound
11	Stuck objects underneath fingernails or into skin
12	Bit parts of my body (e.g. mouth, lips, etc.)
13	Scratched skin until it left scars
14	Picked or peeled off skin
15	Stabbed my body with sharp or pointed objects
Moderate/Severe	
2	Cut my body with sharp objects
7	Cut or carved something onto skin using a knife
10	Burnt skin with fire (using cigarette, match, or other hot objects)
16	Cut holes in my body (e.g., not ear piercings or body piercings that are meant to enhance beauty, but to make holes to harm myself)
17	Slit or cut my body with sharp objects
18	Carved words or symbols onto body
Other	
1	Overdosed on drugs
19	Strangled my neck
20	Engaged in bloodletting (drained blood from my body)
